# Wide Spectrum of Active Compounds in Sea Buckthorn (*Hippophae rhamnoides*) for Disease Prevention and Food Production

**DOI:** 10.3390/antiox10081279

**Published:** 2021-08-12

**Authors:** Agnieszka Jaśniewska, Anna Diowksz

**Affiliations:** Institute of Fermentation Technology and Microbiology, Faculty of Biotechnology and Food Sciences, Lodz University of Technology (TUL), 171/173 Wólczańska Street, 90-924 Łódź, Poland; anna.diowksz@p.lodz.pl

**Keywords:** sea buckthorn, natural antioxidants, bioactive compounds, functional food, nutraceuticals

## Abstract

Growing demand for value-added products and functional foods is encouraging manufacturers to consider new additives that can enrich their products and help combat lifestyle diseases. The healthy properties of sea buckthorn have been recognized for centuries. This plant has a high content of bioactive compounds, including antioxidants, phytosterols, essential fatty acids, and amino acids, as well as vitamins C, K, and E. It also has a low content of sugar and a wide spectrum of volatiles, which contribute to its unique aroma. Sea buckthorn shows antimicrobial and antiviral properties, and is a potential nutraceutical or cosmeceutical. It was proven to help treat cardiovascular disease, tumors, and diabetes, as well as gastrointestinal and skin problems. The numerous health benefits of sea buckthorn make it a good candidate for incorporation into novel food products.

## 1. Introduction

Sea buckthorn is a plant native to China and is found throughout the major temperate zones of the world, including France, Russia, Mongolia, India, Great Britain, Denmark, the Netherlands, Germany, Poland, Finland, and Norway [1]. It can grow under harsh conditions, such as drought, frost, and air pollution [2]. Its generic name, *Hippophae*, derives from the fact that, in ancient Greece, horses were fed with sea buckthorn to give them shiny coats (Greek: *hippos*—horse; *phaos*—shiny) [3,4]. This thorny, dioecious, deciduous shrub with yellow or orange berries belongs to the oleaster family (*Elaeagnaceae*) and can reach a height of 7 m [5,6]. *Hippophae rhamnoides* is divided into nine subspecies based on genetic variations, among which *H. rhamnoides* ssp. *sinensis* Rousi and *H. rhamnoides* ssp. *yunnanensis* Rousi are found only in China [7].

**Figure 1:**
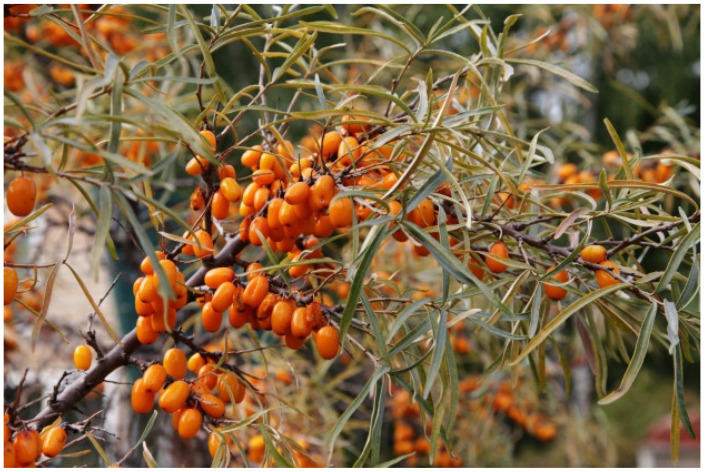
Sea buckthorn branch with berries [11].

Sea buckthorn has long lanceolate leaves with characteristic silver hairs on the underside. It blooms in late April to early May, producing a large number of small green and brown flowers that grow together, forming clusters. In time, round berries, usually yellow or orange in color, are formed. The ripening season is in September. Each fruit contains a small, elongated, grooved stone covering an oily seed [2,6,8,9]. The berries have a characteristic bitter and sour taste with a delicate aroma, resembling a pineapple [2,3,4]. For this reason, sea buckthorn is also known as Siberian pineapple [2]. The dense arrangement of the berries and numerous thorns make harvesting very difficult (Figure 1). For this reason, sometimes entire bunches are removed from the shrub, but this method prevents the growth of later crops [5,6]. As a consequence, berries can be harvested only once every two years [6,10]. In developing countries, especially China, sea buckthorn fruit are still being harvested manually. Other methods of harvesting are direct where vacuum suction harvester is used, or indirect, which is accomplished by shaking a portion of the plant, either by vibration harvesting or cutting harvesting. Forces applied to the trunk or branch result in detaching fruit form the stem [6].

Sea buckthorn contains a variety of bioactive substances, which are present not only in the berries but also in the seeds and leaves [12,13,14,15,16]. Different parts of the plant vary in composition of antioxidants, which has shown a positive biological, physiological, and medicinal effect of sea buckthorn. The most scientific interest focuses on ascorbic acid found in berries, juice, and leaves [17]; phytosterols, such as cycloartenol, campesterol [18]; citrostadienol, sitosterol [19]; carotenoids, among which lycopene, lutein, zeaxanthin, α-carotene, β-carotene, γ-carotene can be found [20,21]; tocopherols—α-tocopherol, β-tocopherol, γ-tocopherol [3]; the most abundant flavonoids, isorhamnetin, and quercetin [22,23], or polyphenolic compounds, such as gallic acid in leaves and berries, and lower amounts of caffeic acid, p-coumaric acid, and ferulic acid [24].

**Figure 2:**
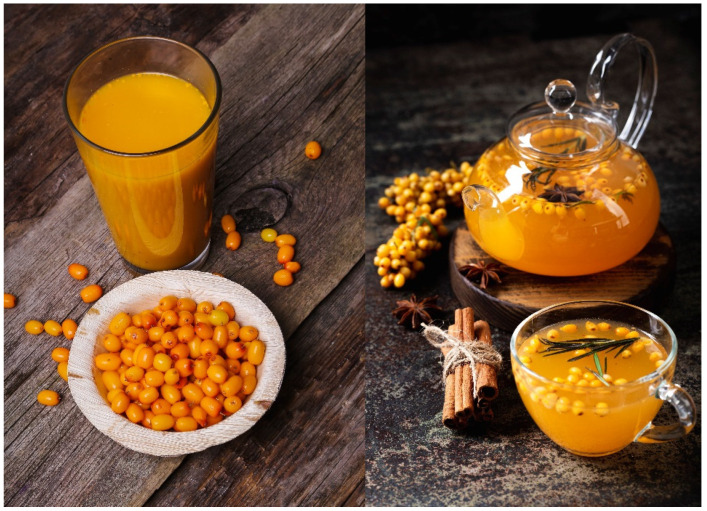
Examples of sea buckthorn in the food industry; juice and tea brewed from berries [31,32].

The plant has been used in traditional medicine since ancient times, in Europe and Asia) [25]. It is widely used in food and feed production (Figure 2). Pressing of the whole fruit results in liquid which after centrifugation can serve as juice. The remaining part is used as oil or oil products [26]. The most popular food prepared from the leaves of sea buckthorn is a tea. This hot beverage is also brewed from berries [27]. Antioxidant-rich jam can be obtained either from sea buckthorn fruit or mixed with other fruit, such as papaya, grapes, or watermelon [28,29]. The use of leaves, seeds, and fruit residues of sea buckthorn have potential as feed material for livestock and poultry in India [30].

Its taste and nutritional properties, together with its health benefits, make sea buckthorn a valuable material for use in a wide range of products, including cosmetics, pharmaceuticals, and food.

## 2. Sea Buckthorn Fruit

Female sea buckthorn plants produce berries that are yellow, orange, or red when ripe. The spherical fruits range in size from 3 to 8 mm [33]. The skin is coated with a thin layer of wax. Inside, each berry contains a single sheathed seed surrounded by a juice-filled cellular structure [34]. The general composition of a sea buckthorn berry is 68% pulp, 23% seed, and 7.75% skin [35,36,37]. Sea buckthorn berries are unpleasant to eat raw, due to their high acidity and astringency. However, several processes can be used to reduce astringency, such as frosting prior to further processing and/or mixing with sweeter juices like apple or grape.

Depending on the subspecies, origin, climate, time of harvesting, and processing methods, the chemical and nutritional composition of sea buckthorn berries can vary [38]. Various kinds of bioactive substances are found in sea buckthorn berries and seed oil) [39]. According to Li and Schroeder and Yao [10], sea buckthorn fruits are one of the most nutritious and vitamin-rich fruits produced by any plant. The nutritional value of sea buckthorn berries surpasses that of other berries since, as well as carbohydrates and proteins, sea buckthorn berries are rich in flavonoids and other antioxidants, fat- or water-soluble vitamins (i.e., vitamins C and E, β-carotene, and lycopene), phytosterols, polyunsaturated fatty acids (especially omega-7 palmitoleic acid), amino acids, and minerals (i.e., iron, calcium, etc.) [26,40]. There are exceptionally large amounts of lipids in sea buckthorn berries compared to other fruits and vegetables that are rich in carotenoids. Lipids enhance the bioavailability of carotenoids, facilitating their absorption in humans [13]. Because of its high content of lipids, the freezing point of unfiltered juice can be as low as −18.5 °C. This is a crucial advantage for storage, as the juice can maintain liquid form even in sub-zero temperatures [41].

### 2.1. Vitamins

The most characteristic feature of sea buckthorn berries is their exceedingly high content of vitamin C. The amount of vitamin C varies from 360 mg/100 g in berries belonging to the *rhamnoides* subspecies, which grows in Europe [42,43,44], to 2500 mg/100 g in berries from the Chinese subspecies *sinensis* [45,46] The amount of vitamin C in fruit grown in the United States ranges from 114 to 1550 mg/100 g, with an average of 695 mg/100 g. This is about 12 times higher than the vitamin C content of oranges. Other fruits commonly considered to be abundant sources of vitamin C, such as strawberries, kiwis, tomatoes, carrots, and hawthorns, also contain much lower concentrations in comparison to sea buckthorn berries [47,48]. The differences between the levels of vitamin C in sea buckthorn berries from different regions are related to the local environmental conditions that occur during the short reproductive period [45]. According to Arimboor [49], the pulp of sea buckthorn berries contains 223.2 mg/100 g of vitamin C. Roughly 75% of the vitamin C in the pulp remains in the juice after processing, resulting in 168.3–184.0 mg/100 g in the final juice.

Other vitamins found in sea buckthorn fruit include vitamin E. The amount of vitamin E in the berries has been measured at 160 mg/100 g [50,51], the amount in the juice as 162–255 mg/100 g [51], which, in the pulp, is at 481 mg/100 g [52], and in the seeds is at 40.1–103.0 mg/100 g [53]. Vitamin K, which is crucial for the post-synthesis modification of proteins that partake in blood coagulation, as well as for controlling calcium binding in bones and other tissues [54], occurs at levels of 110–230 mg/100 g in the berries and in seeds at 109.8–230.0 mg/100 g [55].

### 2.2. Phytosterols

According to Yang and others [38], (the content of total sterols in two subspecies of sea buckthorn (*sinensis* and *rhamnoides*) ranges from 1200–1800 mg/kg in the seeds, 240–400 mg/kg in the fresh pulp/peel, and 340–520 mg/kg in the whole berries. Derivatives of sitosterol were found in the largest quantities. Campesterol, stigmastanol, and R-amyrin were the other major sterols found. The content and composition of these compounds showed slight variations depending on the subspecies and collection sites. The consumption of plant sterols can reduce plasma cholesterols in humans. Lowering cholesterol is important for the treatment of coronary heart disease [33].

### 2.3. Amino Acids

Sea buckthorn berries contain 18 of the 22 known amino acids (Table 1). Amino acids are commonly referred to as the building blocks of proteins. Half of them participate in crucial processes in the human body, such as energy production, building cells and muscles, and fat loss, as well as essential mood and brain functions. Essential amino acids include threonine, valine, methionine, leucine, lysine, tryptophan, isoleucine, and phenylalanine [51].

### 2.4. Organic Acids

The two main organic acids found in sea buckthorn fruits are malic and quinic acids. Together, these acids constitute around 90% of all fruit acids in sea buckthorn plants, although the concentrations vary in different species. Russian berries show relatively lower concentrations of total acidity (2.1–3.2 g/100 mL). The total acidity of Finnish genotypes ranges from 4.2 to 6.5 g/100 mL. Chinese genotypes show the highest concentrations of organic acids, in the range of 3.5–9.1 g/100 mL [51,53,56].

### 2.5. Mineral Elements

The mineral elements that enrich the composition of sea buckthorn fruit include iron, magnesium, copper, zinc, sodium, potassium, phosphorus, calcium, nitrogen, and manganese [57,58]. The most abundant mineral element in the berries and juice is potassium [56], at concentration of 10.12–14.84 ppm in the pulp and 9.33–13.42 ppm in the seed [55]. In a study by Kallio and others, eight elements were compared in berries originating from different countries. Finnish species contained smaller amounts of iron, calcium, and lead but higher levels of cadmium in comparison to Chinese fruit. The maturity of the plant affects the quantity of N, Ca, K, Na, Mg, Cu, Fe, Zn, and Mn [56,59].

### 2.6. Sugars

Glucose, fructose, and xylose are the three main sugars present in sea buckthorn berries. Total soluble sugars in the species grown in China constitute between 5.6% and 22.7% of raw juice [51,53,56,60]. Berries originating from China contained the highest levels of sugars, followed in decreasing order by berries from Russia and Finland) [56]. Yang [61] studied sugar levels in three different subspecies (*Hippophae rhamnoides* ssp. *sinensis*, *rhamnoides*, and *mongolica*) harvested in China, Finland, and Russia over a period of four consecutive years. The combined levels of glucose and fructose varied from 0.6 g/100 mL in the juice from Finnish fruit (ssp. *rhamnoides*) to 24.2 g/100 mL in juice pressed from wild Chinese berries (ssp. *sinensis*). Various levels of sugars were found in different batches. These differences can be explained by the slightly different harvest times and weather conditions in the years during which the crops were grown.

The main sugar present in sea buckthorn berries of all origins is glucose. Together with fructose, glucose accounts for around 90% of the total sugar content in berries harvested in China and Russia. Finnish species are characterized by lower amounts of these sugars (60%) [53,56,62].

### 2.7. Volatile Compounds

Sea buckthorn berries possess a unique aroma, due to their content of volatile compounds. These volatile substances are mainly short chain esters, branched or n-fatty acids, and alcohols. The time of harvest determines the composition of volatiles [38]. Chinese berries contain larger amounts of ethyl 3-methylbutanoate, butyl pentanoate, 2-methylpropyl 3-methylbutanoate, and pentyl 3-methylbutanoate than the Finnish species, which are rich in ethyl 2-methylbutanoate, ethyl 3-methylbutanoate, and ethyl hexanoate [56,63]. In a study by Hirvi and Honkanen [64], 60 volatile compounds were identified using combined gas chromatography-mass spectrometry. The compounds found in the largest quantities were ethyl hexanoate, 3-methylbutyl 3-methylbutanoate, 3-methylbutanoic acid, 3-methylbutyl hexanoate, 3-methylbutyl benzoate, and 3-methylbutyl octanoate. Terpenes and aromatic compounds were found in trace amounts. Cakir, also using combined gas chromatography-mass spectrometry, identified 30 compounds in sea buckthorn volatile oil. The major components were ethyl dodecanoate (39.4%), ethyl octanoate (9.9%), decanol (5.6%), ethyl decanoate (5.5%), and ethyl dodecanoate (3.7%) [65].

## 3. Medicinal Value

**Figure 3:**
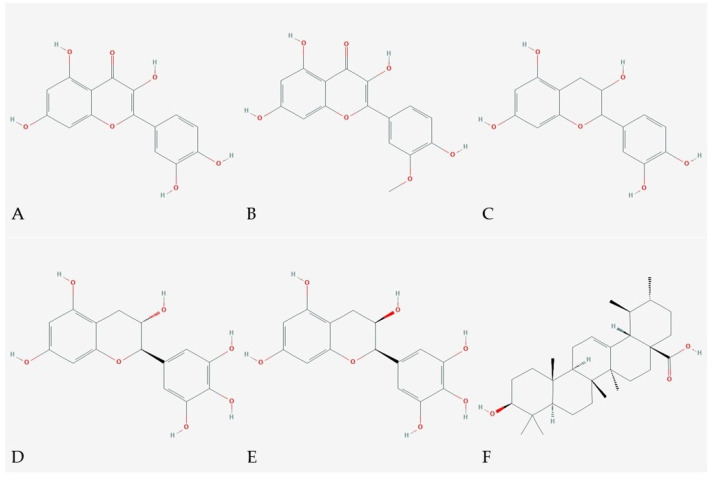
Structures of the most relevant phytochemicals with present in sea buckthorn with medicinal value (**A**)—quercetin, (**B**)—isorhamnetin, (**C**)—catechin, (**D**)—gallocatechin, (**E**)—epigallocatechin, (**F**)—ursolic acid [72,73,74,75,76,77].

Sea buckthorn has been known for its medicinal properties for centuries. Today, it is gaining more attention due to its high nutritional value and wide spectrum of bioactive compounds, which participate in numerous healing processes (Table 2) (Figure 3). The health benefits of the berries include anti-inflammatory effects, antimicrobial action, pain relief, promotion of tissue regeneration, and boosting of the immune system, as well as protection against cancer and cardiovascular disease. Sea buckthorn has found application in many medical preparations, aimed at treating cancer, heart ailments, ulcers, hepatic disorders, burns, and brain disorders [66].

**Table 2:** Major phytochemicals in sea buckthorn and their medicinal properties.

Sea Buckthorn Phytoconstituents	Medicinal Properties	References
**Tocopherols**	Act as antioxidants; minimize lipid oxidation; help to relieve pain	[3]
**Carotenoids**	Act as antioxidants; help in collagen synthesis and epithelialization	[67]
**Vitamin K**	Prevents bleeding; promotes wound healing; shows anti-ulcer effects	[68]
**Vitamin C**	Acts as an antioxidant; sustains cell membrane integrity; accelerates collagen synthesis	[3]
**Vitamin B complex**	Stimulates cell repair and nerve regeneration	[68]
**Phytosterols**	Improve microcirculation in the skin; show anti-ulcer, anti-atherogenic, and anti-cancer effects; regulate inflammatory processes	[38]
**Polyphenolic compounds**	Show antioxidant, cytoprotective, and cardioprotective effects; promote wound healing	[69]
**Poly unsaturated fatty acids (PUFA)**	Immunomodulatory, neuroprotective, and anti-tumor activity	[36]
**Organic acids**	Lower the risk of heart attack and stroke; show anti-ulcer effects; promote wound healing; anti-arthritic	[36]
**Coumarins and triterpenes**	Control of appetite; promote sleep, memory, and learning	[70]
**Zinc**	Strengthens the blood circulation; aids in cell proliferation; reveals anti-tumor effects; acts as a cofactor for enzymes; enhances utilization of vitamin A	[71]

### 3.1. Mineral Elements

The antioxidant activity of sea buckthorn fruit extracts can be attributed to their ability to capture free radicals. Alcoholic extract of dried sea buckthorn berries has been found to be a more effective antioxidant than the standard antioxidants 2,6-di-tert-butyl-p-hydroxytoluene (BHT) and tert-butyl-hydroxyanisole (BHA) [78]. In a study by Varshneya et al. [79], different sea buckthorn extracts were evaluated in terms of antioxidant activity. The reducing power of the extracts increased in a dose-dependent manner and was highest in 70% methanol extract. Alcoholic fruit extract of sea buckthorn showed significant cytoprotection against sodium nitroprusside-induced oxidative stress in lymphocytes [80].

The antioxidant and immunomodulatory properties of sea buckthorn have been studied in vitro using rat splenocytes, macrophages, and the C-6 glioma cell line, as well as in vivo using male albino rats. Alcoholic leaf extract of sea buckthorn (500 g/mL) was found to inhibit the production of chromium-induced free radicals, as well as apoptosis, and to restore antioxidant status to that in control cells [81]. Even at a lower concentration (100 mg/kg), the extract protects rats from chromium-induced oxidative damage [82]. The leaf extract also has the ability to protect glial cells against hypoxia-induced oxidative damage [83].

### 3.2. Cardiovascular Diseases

Flavonoids are polyphenols that occur naturally in fruits and vegetables, including sea buckthorn. The most abundant flavonoids in sea buckthorn fruit and leaves are isorhamnetin and quercetin [22]. The antioxidant properties of flavanols have been reported to lower the risk of cardiovascular diseases. Total sea buckthorn flavonoids show protective effects against myocardial ischemia and reperfusion, tumors, oxidative injury, and aging [84]. Flavonoids from sea buckthorn protect endothelial cells from oxidized low-density lipoprotein induced injuries [85]. Hypertensive stroke-prone rats fed ad libitum with 0.7 g/kg dried sea buckthorn fruit powder for 60 days showed improved metabolic processes and reduced hypertensive stress [86].

In a study by Pang and others [87], rats were administered with food high in sucrose, which significantly increased their systolic blood pressure, as well as the levels of insulin and triglycerides in their plasma, and the amount of angiotensin II in their heart and kidneys. The experimental group was then given a diet enriched with sea buckthorn seed extract. The results showed antihypertensive action by blocking the angiotensin II pathway and improving sensitivity to insulin. Similarly, rabbits on a high cholesterol diet given 1 mL of sea buckthorn seed oil per day for 30 days had reduced LDL, a lower atherogenic index and showed increased HDL and vasorelaxant activity [88]. In a study by Johansson et al. [89], 12 healthy normolipidemic men were given 5 g of sea buckthorn oil a day for a month. Their levels of phospholipid fatty acids, plasma lipids, and glucose were unaffected. Instead, a clear decrease was observed in the rate and extent of adenosine-5′-diphosphate-induced platelet aggregation. Blood clotting is considered one of the most important risk factors for cardiovascular disease.

Even though polyphenols are the main focus of many studies involving prevention of development of cardiovascular diseases, probiotics have been proven to enhance the nutrients’ bioavailability and shown reduction of the risk of developing various health conditions including cardiovascular diseases. A possible mechanism of action of probiotics is due to the inhibition of hepatic lipogenesis and in the same time decreasing the glucose level in blood and insulinemia [90,91,92]. Resveratrol together with quercetin have been connected with enhancement of physiological functionality of *Lactobacillus* strains. These improvements depend on the type and concentration of the compound used as well as the bacteria strain. Quercetin showed better protective effects than resveratrol and among the tested strains, the best results were obtained for *L. fermentum* and *L. plantarum* strains [91].

### 3.3. Diabetes

Diabetes is a metabolic disorder of the endocrine system. It prevents patients from being able to properly produce and use insulin in the body, resulting in high content of blood glucose [93]. Numerous researchers have examined the potential of sea buckthorn to aid in the treatment of diabetes. Zhang and others [94] investigated the effects of an aqueous extract of sea buckthorn seed residues on serum glucose, lipid profiles, and antioxidant parameters in streptozotocin-induced diabetic rats. Four groups of rats were examined: a normal control group, a diabetic control group, a diabetic group supplemented with 5 mg/kg body weight of glibenclamide (reference drug), and another a diabetic group supplemented with 400 mg/kg body weight of sea buckthorn seed residue extract. The extract significantly lowered the levels of serum glucose, triglyceride, and nitric oxide in the diabetic rats. Moreover, there were noticeable increases in serum superoxide dismutase activity and levels of glutathione. This demonstrates the potential hypoglycemic, hypotriglyceridemic, and antioxidant effects of sea buckthorn supplements, suggesting that sea buckthorn could be useful for preventing diabetic complications associated with hyperlipidemia and oxidative stress.

Kim et al. [95] studied the antioxidant activity of extracts, fractions, and isolated compounds of sea buckthorn leaves, as well as their ability to inhibit α-glucosidase. Six compounds, kaempferol-3-O-β-D-(6′-O-coumaryl) glycoside, 1-feruloyl-β-D-glucopyranoside, isorhamnetin-3-O-glucoside, quercetin-3-O-β-D-glucopyranoside, quercetin-3-O-β-D-glucopyranosyl-7-O-α-L-rhamnopyranoside, and isorhamnetin-3-O-rutinoside, were isolated from the extracts. The butanol fraction, which contained the largest amounts of phenolic compounds, showed the highest radical-scavenging activity and also the most powerful α-glucosidase inhibitory effect.

The positive effects of sea buckthorn can be enhanced by their combination with other berries. An improvement in blood sugar and lipid levels was reported in children with type I diabetes who were fed blueberry and sea buckthorn concentrate formulae. This improvement might be due to the combined beneficial effects of the berries. Combinations of these two species could also be effective at preventing cardiovascular diseases, diabetes, and related complications [96]. In another study focusing on the combined effects of sea buckthorn and blueberry concentrate, 30 type I diabetic children were given two-berry concentrates as dietary supplements. The results showed a significant decrease in glycated hemoglobin and an increase in the concentration of C peptides [97].

Again, not only polyphenols themselves, but also their bioavailability matters in the natural treatment for type 2 diabetes. The issue is tightly correlated with the way polyphenols influence the gut microbiota. It depends on their chemical structure, the matrix they might be a part of, a dosage that is introduced into the system or the type of diet [92]. Polyphenol-rich extracts that have been proven effective in lowering the blood glucose levels have also been shown to affect the gut microbiota [98]. Their synergistic effect resulted in the reduction of *Firmicutes* abundance, the downstream of *Clostridiales* and *Lachnospiraceae*, and a high concentration of butyrate. A study by Roopchand and others [99] showed that dietary polyphenols lowered intestinal expression of inflammatory markers and a gene for glucose absorption (Glut2) as well as modified the structure of gut microflora in mice by increasing the growth of *Akkermansia muciniphila* in the same time decreasing the proportion of *Firmicutes* to *Bacteroidetes*.

### 3.4. Antitumor Effect

The antitumor activity of extract of sea buckthorn has been demonstrated using an initiator (7,12-dimethylbenz[a]anthracene) and a promoter (12-O-tetradecanoylphorbol-13-acetate (TPA)) of carcinogenesis in mice. Three phenolic compounds (catechin, gallocatechin, and epigallocatechin) and a triterpenoid (ursolic acid) isolated from sea buckthorn were shown to prevent TPA-induced inflammation [100].

The cytotoxic effects of sea buckthorn flavonoids have also been studied in human hepatocellular carcinoma cells (BE2-7402) [101]. Cytotoxic action was observed, caused by the accumulation of isorhamnetin in cells. After 48 h of treatment, the BE2-7402 tumor cells exhibited isorhamnetin-induced chromatin condensation and fragmentation, indicating that the sea buckthorn extract exerted antitumor and growth inhibitory effects on the tumor cells.

Kim and others [102] examined whether sea buckthorn leaf extract inhibited proliferation and promoted apoptosis in rat glioma C6 cells. Treatment with sea buckthorn leaf extract inhibited the proliferation of rat C6 glioma cells in a dose-dependent manner and decreased production of reactive oxygen species, which are critical for the proliferation of tumor cells. Sea buckthorn treatment not only upregulated the expression of pro-apoptotic protein Bcl-2-associated X (Bax) significantly, but also promoted its localization in the nucleus.

A study by Nersesyan and Muradyan [103] investigated the influence of sea buckthorn juice on micronucleus frequency in bone marrow cells and sperm abnormality induced by cisplatin. At a dose of 1.2 mg/kg, the sea buckthorn juice caused a significant decrease in the genotoxic effect of cisplatin on somatic (bone marrow) and germ (sperm) cells in mice.

Sea buckthorn extract contains β-sitosterol, which have also been reported to exert antitumor activity [104,105,106].

### 3.5. Gastrointestinal Effect

Sea buckthorn has been investigated for its ability to treat diseases of the gastrointestinal tract [107]. Groups of rats with induced gastric ulcers were subjected to pre-trial oral administration of CO_2_-extracted seed and pulp oil. Both the protective and curative effects of the sea buckthorn oils were studied. Sea buckthorn oil intake accelerated the healing process of acetic acid-induced gastric ulcers. In other research on rats, ulcers induced in the same way were treated with procyanidins extracted from the sea buckthorn oil [108]. Reductions in the sizes of the ulcers were noticed on days 7 and 14 in a dose-dependent manner. This suggests that the procyanidins in sea buckthorn play an important role in healing acetic acid-induced gastric lesions, possibly by accelerating mucosal repair. A study by Suleyman and others [109] examined stress- and indomethacin-induced gastric ulcers in rats. Sea buckthorn supplementation significantly reduced the size of the induced ulcers, and in some cases only hyperemia or even no ulcer was found.

A crucial component of the gastrointestinal tract is its natural microflora. The effect of sea buckthorn on beneficial bacteria was taken under investigation. In Hao’s study [19], it was found that the supplementation of hamster feed with sea buckthorn seed oil could enhance the production of fecal short-chain fatty acids, especially acetic acid and butyric acid. Another mechanism resulting in lowering blood cholesterol by sea buckthorn seed oil is possibly mediated by stimulation of the growth of microbiota that produces short-chain fatty acids. The results showed an increase in the abundance of the short-chain fatty acids-generating Bacteroidales S24-7 group and decrease in *Ruminococcaceae*.

Attri and Goel [110] focused on the effect of polyphenol rich sea buckthorn berries juice on colonic microbial composition and diversity using in vitro simulated gut model. The study indicated the stimulatory effect of sea buckthorn juice on the beneficial microbial population of *Lactobacilli*, *Bacteroides/Prevotella* and *Bifidobacteria*. Higher content of resveratrol, rutin, and chlorogenic acid were observed in ascending colon, whereas quercetin, ferulic, and caffeic acid levels were higher in the descending colon due to biotransformation of polyphenols in the later part of the colon.

Another study by Yuan [111] investigated the effect of sea buckthorn protein on intestinal microbial community in streptozotocin-induced diabetic mice. It was found that sea buckthorn protein can increase the number of *Bifidobacterium, Lactobacillus,* and *Bacteroides* and reduce the number of *Clostridium coccoides*. Previous research stated that *Bifidobacterium, Lactobacillus,* and *Bacteroides* act as beneficial bacteria that can positively affect the immune system of the host by promotion of macrophage activity, enhancement of B and T lymphocytes to increase the reactivity of the antigen, stimulation of the thymus, spleen, and other immune organs [112,113]. Apart from positive effects on gut microflora, sea buckthorn protein served in the form of a natural food additive might be beneficial in a therapeutic diet for diabetic patients.

### 3.6. Wound Healing

Sea buckthorn has traditionally been used to aid skin regeneration, and has also found applications in modern medicine. Sea buckthorn fruit and seed oil contains high levels of beneficial unsaturated fatty acids (omega-3,6,7), natural antioxidants, vitamins (E, K), carotenoids, and phytosterols [26]. All of these chemicals combine to protect cell membranes and enhance cell regeneration. Palmitoleic acid is a component of skin used in burn treatment and wound healing. Sea buckthorn oil has been reported to have preventive and curative effects against different types of gastric ulcers, chronic cervicitis, and atopic dermatitis [107,114].

Edraki and others [115] treated burns on rats with sea buckthorn oil and olive oil, separately and as a mixture. Both the sea buckthorn and the olive oil were effective dressings for burn wounds; but together they showed a synergetic effect. The group treated with the sea buckthorn/olive oil mixture showed more developed re-epithelialization, with a continuous basement membrane and mature granulation tissue.

Sea buckthorn oil has been tested for its potential to ease the symptoms of menopause, such as vaginal atrophy and the thinning and drying of vaginal mucosa. It is suitable for women who cannot tolerate estrogen treatment. In one study, over 90 women were given sea buckthorn oil supplements, administered orally [116]. After three months, the women showed improved vaginal health, including significantly better integrity of the vaginal epithelium. Treatment with sea buckthorn oil can therefore offer an alternative for estrogen therapy for vaginal atrophy.

Given the known positive effects of sea buckthorn oil on skin and mucous membranes, researchers have investigated its possible effects on dry eye syndrome. A group of men and women with dry eye syndrome were given sea buckthorn oil, administered orally, over a period of three months in autumn and winter. The sea buckthorn oil attenuated the increase in tear film osmolarity during the cold season and positively affected dry-eye symptoms [117].

Alcoholic sea buckthorn leaf extract was shown to upregulate the antigen presentation of macrophages in aged mice, with immune-boosting and anti-aging effects [118].

### 3.7. Antimicrobial and Antiviral Properties

Sea buckthorn possesses antiviral and antimicrobial properties. Chaman and others [119] used the hole-plate diffusion method to test the antibacterial potential of sea buckthorn berry extracts. The results showed that methanol extract (100 mg/mL) produced a comparatively marked antibacterial response, whereas other extracts showed a weak zone of inhibition against all types of the tested bacteria. Smida and others [120] designed a mouthwash based on sea buckthorn pulp oil, which they compared with two commercially available mouthwash products and evaluated against *Streptococcus gordonii, Porphyromonas gingivalis, Actinomyces viscosus*, and *Candida albicans*. The experimental preparation was bactericidal against *S. gordonii* and *P. gingivalis*, bacteriostatic against *A. viscosus*, and showed no antifungal effect. Irrespective of the tested strains, complete inhibition of biofilm formation was achieved.

Sea buckthorn also exhibits an antiviral response to Dengue virus infection. Dengue fever is a serious condition with no direct treatment. *H. rhamnoides* leaf extract was tested in Dengue virus type-2 infected blood-derived human macrophages and compared with the commercially available anti-viral drug Ribavirin. The extract was equally effective at maintaining the cell viability of Dengue-infected cells as Ribavirin, suggesting that *H. rhamnoides* leaf extract has significant anti-dengue activity and could be used to treat Dengue fever [121].

Other studies have investigated the antimicrobial properties of *H. rhamnoides* extracts against microorganisms that cause serious food poisoning and infections. Methanol extracts and fractions of the root and stem of sea buckthorn showed better antimicrobial activity than the antimicrobial agents (+)-catechin, ketoconazole, and mycostatin, especially against *C. albicans*, *P. jadinii*, *B. subtilis*, and *S. aureus*. This points towards possible applications of sea buckthorn in the food industry, as an additive and for the development of useful natural compounds [122].

## 4. Food Applications

Sea buckthorn is already applied in numerous food products. The most popular products prepared from sea buckthorn are juices, jams, wines, pies, and liquors. Due to their high acidity, the fruits can be used to make refreshing drinks, while the leaves are used in teas [26,123]. Despite broad beneficial health effects sea buckthorn fruit might be unpleasant to eat raw, due to the high acidity and astringency. Studies focus on different compounds that are responsible for unpleasant taste of the berry. Malic and quinic acids are the main representatives among organic acids in sea buckthorn fruit which contribute to pungency. To avoid that, some producers use malolactic acid fermentation, a traditional technique used in winemaking, to lower its content by converting it to milder lactic acid [124]. Another substance responsible for lowering pleasantness is ethyl-β-D-glucosidase. Overripe juice tend to poses higher content of ethyl-β-D-glucosidase, which results in a significantly more bitter taste and causes lower notes in sensory panel tasting [125]. Ratios between contents of various compounds impact astringency and bitterness more significantly than the amounts of individual variables, such as proanthocyanidin dimers and trimers or quercetin glycosides, which are known for heightening perception of astringent taste [126].

Sea buckthorn has been supplied to Russian cosmonauts. In the Seoul Olympics in 1992, sea buckthorn squash was the official health drink of the Chinese athletes [127].

The rich spectrum of bioactive compounds found in sea buckthorn has inspired researchers to investigate the application of its various parts and extracts in food. Terpou and others [128] combined the nutritional properties of *H. rhamnoides* with the gut-boosting abilities of probiotic bacteria. Feta cheese was used as a carrier of *L. casei* immobilized on sea buckthorn berry. The result was a product with improved physicochemical properties, an aroma enriched with terpenes and carbonyl compounds, and with a higher probiotic cell population. The same bacteria strain was used to fortify frozen yoghurt, with sea buckthorn berries as the immobilization carrier. Sea buckthorn berries, in addition to providing microbiological safety, imparted exceptional sensory features [129].

Lactose intolerant consumers have limited access to probiotic products. Maftei and others [39] developed a soy drink with sea buckthorn syrup, fermented by probiotic *L. casei*. Increasing the concentration of the sea buckthorn syrup caused an increase in the viable cell count, but also raised the acidity and lowered the pH during fermentation at 30 °C or 37 °C.

The meat industry is currently looking for natural additives to replace artificial supplements. In one study, sea buckthorn berry powder and brews were incorporated as ingredients in pork sausages. Fortification with sea buckthorn berry powder strongly inhibited lipid oxidation during storage, prolonging shelf life [130].

Sea buckthorn has also been applied in alcoholic beverages. Sea buckthorn wine has been found to exert a protective effect in mice against phorone-induced oxidative stress and hypercholesterolemia induced by a high-cholesterol diet [131]. Reduced hepatic lipid peroxidation and increased superoxide dismutase activity were also observed. The results of high-performance liquid chromatographic analysis showed that the wine contained high levels of rutin, myricetin, and quercetin compared with commercially available Cabernet Shiraz wine. In another study, Wang and others [132] identified the volatile compounds in sweet and dry sea buckthorn wine, raw juice, and fermented must, which contained 53, 48, 37, and 38 compounds, respectively. These compounds contributed to the specific taste of the sea buckthorn wine. Sea buckthorn beer has also been the focus of research. In one study, fruit mash was added after four weeks of fermentation to green beer, resulting in good technological parameters. In total, 32 volatile substances were identified, enriching the flavor and aroma. Higher antiradical DPPH activity was observed compared to the control [133].

## 5. Conclusions

Sea buckthorn is a rich source of many active substances with health promoting properties. This wide spectrum of bioactive compounds can help prevent or treat a range of conditions, such as cardiovascular disease, diabetes, tumors, gastrointestinal disorders, and skin problems. Sea buckthorn is therefore an excellent supplement with which to enrich the daily diet, helping to prevent diseases of affluence. Moreover, sea buckthorn shows synergetic effects when combined with other plants with pro-health properties. The antimicrobial and antiviral properties of sea buckthorn make it suitable for use in the production of pharmaceuticals targeted against specific strains of microorganisms. Due to its antioxidant properties and high nutritional value, sea buckthorn has already found numerous applications in foods, beverages, supplements, and medicines, and many more potential uses are being investigated.

## Figures and Tables

**Table 1:** Amino acids found in sea buckthorn according to [51].

Amino Acid	Content (mg/100 g)
**Aspartic acid**	426.6
**Serine**	28.1
**Glutamine**	19.4
**Glycine**	16.7
**Alanine**	21.2
**Cysteine**	3.3
**Valine**	21.8
**Ammonia**	41.8
**Tyrosine**	13.4
**Isoleucine**	17.4
**Methionine**	2.3
**Proline**	45.2
**Phenylalanine**	20.0
**Histidine**	13.7
**Lysine**	27.2
**Threonine**	36.8
**Arginine**	11.3
